# Poly[μ-aqua-[μ-1,1′-(butane-1,4-di­yl)diimidazole]bis­(μ_4_-naphthalene-1,4-dicarboxyl­ato)dicadmium(II)]

**DOI:** 10.1107/S1600536808037525

**Published:** 2008-11-20

**Authors:** Qun Xu, Wen-Zhi Zhang, Zhi-Qiang Chen

**Affiliations:** a College of Chemistry and Chemical Engineering, Qiqihar University, Qiqihar 161006, Heilongjiang Province, People’s Republic of China

## Abstract

In the title compound, [Cd_2_(C_12_H_6_O_4_)_2_(C_10_H_14_N_4_)(H_2_O)]_*n*_, the coordination polyhedron around each Cd^II^ ion is a distorted CdNO_5_ octa­hedron. The water O atom has site symmetry 2 and the complete 1,1′-(butane-1,4-di­yl)diimidazole (*L*) ligand is generated by inversion. The naphthalene-1,4-dicarboxyl­ate and *L* ligands bridge the metal centres, forming a three-dimensional framework, which is consolidated by O—H⋯O hydrogen bonds.

## Related literature

For background to metal–organic frameworks, see Ma *et al.* (2003 ▶); Yang *et al.* (2008 ▶).
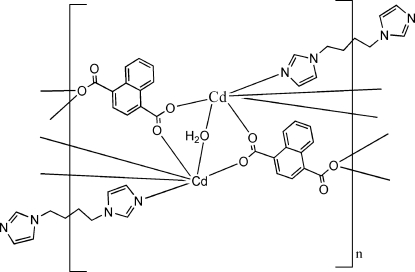


## Experimental

### 

#### Crystal data

[Cd_2_(C_12_H_6_O_4_)_2_(C_10_H_14_N_4_)(H_2_O)]
*M* *_r_* = 861.40
Monoclinic, 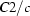

*a* = 18.773 (2) Å
*b* = 14.9118 (19) Å
*c* = 14.2298 (18) Å
β = 127.3900 (10)°
*V* = 3165.0 (7) Å^3^
*Z* = 4
Mo *K*α radiation
μ = 1.41 mm^−1^
*T* = 293 (2) K
0.33 × 0.27 × 0.22 mm

#### Data collection

Bruker APEX CCD diffractometer
Absorption correction: multi-scan (*SADABS*; Bruker, 1998 ▶) *T*_min_ = 0.691, *T*_max_ = 0.732
8715 measured reflections
3102 independent reflections
2809 reflections with *I* > 2σ(*I*)
*R*_int_ = 0.018

#### Refinement

*R*[*F* ^2^ > 2σ(*F* ^2^)] = 0.022
*wR*(*F* ^2^) = 0.056
*S* = 1.06
3102 reflections
226 parameters
H atoms treated by a mixture of independent and constrained refinement
Δρ_max_ = 0.47 e Å^−3^
Δρ_min_ = −0.30 e Å^−3^

### 

Data collection: *SMART* (Bruker, 1998 ▶); cell refinement: *SAINT* (Bruker, 1998 ▶); data reduction: *SAINT*; program(s) used to solve structure: *SHELXS97* (Sheldrick, 2008 ▶); program(s) used to refine structure: *SHELXL97* (Sheldrick, 2008 ▶); molecular graphics: *SHELXTL* (Sheldrick, 2008 ▶); software used to prepare material for publication: *SHELXTL*.

## Supplementary Material

Crystal structure: contains datablocks global, I. DOI: 10.1107/S1600536808037525/hb2837sup1.cif

Structure factors: contains datablocks I. DOI: 10.1107/S1600536808037525/hb2837Isup2.hkl

Additional supplementary materials:  crystallographic information; 3D view; checkCIF report

## Figures and Tables

**Table 1:** Selected bond lengths (Å)

Cd1—N1	2.264 (2)
Cd1—O2	2.2746 (17)
Cd1—O1^i^	2.2344 (17)
Cd1—O4^ii^	2.3096 (16)
Cd1—O4^iii^	2.4847 (15)
Cd1—O1*W*	2.2995 (14)

Symmetry codes: (i) 
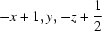
; (ii) 
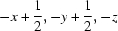
; (iii) 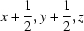.

**Table 2:** Hydrogen-bond geometry (Å, °)

*D*—H⋯*A*	*D*—H	H⋯*A*	*D*⋯*A*	*D*—H⋯*A*
O1*W*—H*W*11⋯O3^iii^	0.76 (3)	1.80 (3)	2.549 (2)	169 (3)

Symmetry code: (iii) 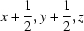.

**Fig. 1.**
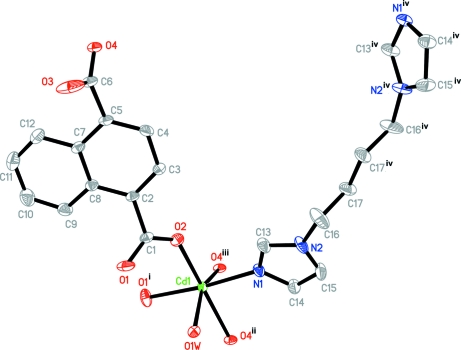
The asymmetric unit of (I), extended to show the Cd coordination sphere and the complete L ligand. Displacement ellipsoids are drawn at the 30% probability level and H atoms are omitted for clarity. Symmetry code: (i) 1-x, y, 0.5-z; (ii) x+0.5, y+0.5, z; (iii) 0.5-x, 0.5-y, -z; (iv) -x, y, -0.5-z.

**Fig. 2.**
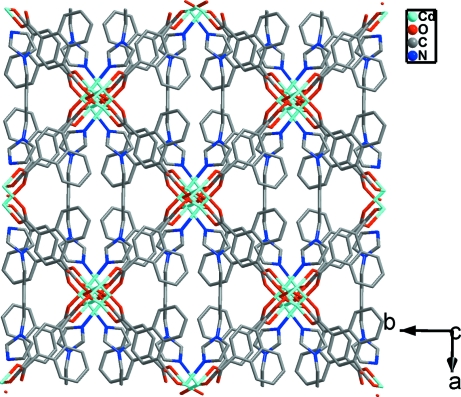
View of the three-dimensional framework of (I).

## supplementary crystallographic information

### Comment

Currently, metal-organic frameworks are of great interest because of their interesting structures and potential applications.  Up to now, some interesting interpenetrated or entangled metal-organic networks with bis(imidazole)-containing ligands have been documented (Yang *et al.*,  2008).   However, flexible ligands such as 1,1'-(butane-1,4-diyl)diimidazole (*L*) has not been well explored to date (Ma *et al.*, 2003). In this work, we selected naphthalene-1,4-dicarboxylic acid (1,4-H_2_ndc) and *L* as linkers in combination with a source of cadmium ions, generating a new coordination polymer, [Cd_2_(1,4-ndc)_2_(*L*)(H_2_O)], (I), which is reported here.

In compound (I) each Cd^II^ atom is six-coordinated by one N atom from one *L* ligand, and five O atoms from four carboxylate oxygen atoms and one water molecule in a distorted octohedral coordination sphere (Fig. 1, Table 1). The water molecule O atom has site symmetry 2 and the *L* ligand is situated across an inversion centre.  The two neighbouring Cd^II^ atoms are bridged by the carboxylate and water molecule to form a dimer. The dimers are further linked by the backbone of 1,4-ndc and *L* ligands to form a three-dimensional framework (Fig. 2).  An O—H···O hydrogen bond (Table 2) helps to consolidate the packing.

### Experimental

A mixture of 1,4-H_2_ndc (0.5 mmol), *L* (0.5 mmol), NaOH (1 mmol) and CdCl_2_^.^2.5H_2_O (0.5 mmol) was suspended in 14 ml of deionized water and sealed in a 20-ml Teflon-lined autoclave. Upon heating at 413 K for three days, the autoclave was slowly cooled to room temperature. The resulting colourless blocks of (I) were collected, washed with deionized water and dried.

### Refinement

The C–bound H atoms were positioned geometrically (C—H = 0.93–0.96 Å) and refined as riding, with *U*_iso_(H) = 1.2*U*_eq_(carrier). The water H atom was located in a difference Fourier map and refined freely.

### Crystal data

**Table:**

[Cd_2_(C_12_H_6_O_4_)_2_(C_10_H_14_N_4_)(H_2_O)]	*F*_000_ = 1712
*M**_r_* = 861.40	*D*_x_ = 1.808 Mg m^−^^3^
Monoclinic, *C*2/*c*	Mo *K*α radiation λ = 0.71073 Å
Hall symbol: -C 2yc	Cell parameters from 3102 reflections
*a* = 18.773 (2) Å	θ = 1.1–26.0º
*b* = 14.9118 (19) Å	µ = 1.41 mm^−^^1^
*c* = 14.2298 (18) Å	*T* = 293 (2) K
β = 127.3900 (10)º	Block, colorless
*V* = 3165.0 (7) Å^3^	0.33 × 0.27 × 0.22 mm
*Z* = 4	

### Data collection

**Table:**

Bruker APEX CCD diffractometer	3102 independent reflections
Radiation source: fine-focus sealed tube	2809 reflections with *I* > 2σ(*I*)
Monochromator: graphite	*R*_int_ = 0.018
*T* = 293(2) K	θ_max_ = 26.0º
ω scans	θ_min_ = 1.9º
Absorption correction: multi-scan(SADABS; Bruker, 1998)	*h* = −16→23
*T*_min_ = 0.691, *T*_max_ = 0.732	*k* = −18→17
8715 measured reflections	*l* = −16→17

### Refinement

**Table:**

Refinement on *F*^2^	Secondary atom site location: difference Fourier map
Least-squares matrix: full	Hydrogen site location: inferred from neighbouring sites
*R*[*F*^2^ > 2σ(*F*^2^)] = 0.022	H atoms treated by a mixture of independent and constrained refinement
*wR*(*F*^2^) = 0.056	*w* = 1/[σ^2^(*F*_o_^2^) + (0.0287*P*)^2^ + 3.5082*P*] where *P* = (*F*_o_^2^ + 2*F*_c_^2^)/3
*S* = 1.07	(Δ/σ)_max_ = 0.001
3102 reflections	Δρ_max_ = 0.47 e Å^−^^3^
226 parameters	Δρ_min_ = −0.30 e Å^−^^3^
Primary atom site location: structure-invariant direct methods	Extinction correction: none

### Special details

**Table:**

Geometry. All esds (except the esd in the dihedral angle between two l.s. planes) are estimated using the full covariance matrix. The cell esds are taken into account individually in the estimation of esds in distances, angles and torsion angles; correlations between esds in cell parameters are only used when they are defined by crystal symmetry. An approximate (isotropic) treatment of cell esds is used for estimating esds involving l.s. planes.
Refinement. Refinement of F^2^ against ALL reflections. The weighted R-factor wR and goodness of fit S are based on F^2^, conventional R-factors R are based on F, with F set to zero for negative F^2^. The threshold expression of F^2^ > 2sigma(F^2^) is used only for calculating R-factors(gt) etc. and is not relevant to the choice of reflections for refinement. R-factors based on F^2^ are statistically about twice as large as those based on F, and R- factors based on ALL data will be even larger.

### Fractional atomic coordinates and isotropic or equivalent isotropic displacement parameters (Å^2^)

**Table:**

	*x*	*y*	*z*	*U*_iso_*/*U*_eq_	
C1	0.37567 (16)	0.35138 (16)	0.1979 (2)	0.0297 (5)	
C2	0.30731 (16)	0.28246 (16)	0.1737 (2)	0.0280 (5)	
C3	0.21801 (17)	0.30336 (18)	0.0966 (2)	0.0359 (6)	
H3	0.2005	0.3550	0.0509	0.043*	
C4	0.15233 (17)	0.24857 (19)	0.0850 (2)	0.0374 (6)	
H4	0.0922	0.2646	0.0328	0.045*	
C5	0.17624 (16)	0.17188 (17)	0.1500 (2)	0.0305 (5)	
C6	0.10956 (17)	0.12334 (17)	0.1573 (2)	0.0333 (6)	
C7	0.26737 (17)	0.14297 (16)	0.2231 (2)	0.0300 (5)	
C8	0.33359 (16)	0.19901 (16)	0.2353 (2)	0.0288 (5)	
C9	0.42311 (18)	0.16783 (19)	0.3048 (2)	0.0422 (6)	
H9	0.4676	0.2031	0.3133	0.051*	
C10	0.4447 (2)	0.0865 (2)	0.3594 (3)	0.0579 (9)	
H10	0.5038	0.0670	0.4048	0.069*	
C11	0.3796 (3)	0.0319 (2)	0.3485 (3)	0.0549 (9)	
H11	0.3957	−0.0234	0.3864	0.066*	
C12	0.2933 (2)	0.05926 (19)	0.2830 (3)	0.0432 (7)	
H12	0.2505	0.0229	0.2771	0.052*	
C13	0.26154 (18)	0.5557 (2)	−0.0295 (3)	0.0433 (7)	
H13	0.2482	0.5072	−0.0015	0.052*	
C14	0.32799 (18)	0.64237 (19)	−0.0731 (2)	0.0404 (6)	
H14	0.3701	0.6657	−0.0810	0.048*	
C15	0.2503 (2)	0.6826 (2)	−0.1106 (3)	0.0471 (7)	
H15	0.2295	0.7378	−0.1484	0.056*	
C16	0.1209 (2)	0.6416 (3)	−0.1088 (3)	0.0654 (10)	
H16A	0.1215	0.6989	−0.0761	0.078*	
H16B	0.1111	0.5954	−0.0697	0.078*	
C17	0.04549 (17)	0.6405 (2)	−0.2362 (3)	0.0479 (7)	
H17A	0.0508	0.5876	−0.2712	0.057*	
H17B	0.0502	0.6926	−0.2729	0.057*	
N1	0.33508 (13)	0.56210 (15)	−0.02170 (18)	0.0332 (5)	
N2	0.20866 (14)	0.62697 (18)	−0.0825 (2)	0.0440 (6)	
O1	0.43795 (13)	0.36693 (13)	0.30491 (17)	0.0455 (5)	
O2	0.36314 (12)	0.38808 (13)	0.11038 (17)	0.0438 (5)	
O1W	0.5000	0.55213 (17)	0.2500	0.0267 (5)	
O3	0.11724 (18)	0.13705 (18)	0.2487 (2)	0.0756 (9)	
O4	0.05171 (10)	0.07264 (11)	0.07468 (14)	0.0285 (4)	
Cd1	0.453957 (10)	0.468475 (11)	0.086231 (14)	0.02277 (7)	
HW11	0.5363 (19)	0.581 (2)	0.257 (3)	0.047 (9)*	

### Atomic displacement parameters (Å^2^)

**Table:**

	*U*^11^	*U*^22^	*U*^33^	*U*^12^	*U*^13^	*U*^23^
C1	0.0285 (13)	0.0274 (13)	0.0399 (14)	−0.0057 (10)	0.0242 (12)	−0.0041 (10)
C2	0.0297 (13)	0.0301 (13)	0.0297 (12)	−0.0090 (10)	0.0210 (11)	−0.0043 (9)
C3	0.0322 (14)	0.0360 (14)	0.0372 (14)	−0.0070 (11)	0.0199 (12)	0.0061 (11)
C4	0.0224 (13)	0.0495 (16)	0.0335 (13)	−0.0073 (11)	0.0135 (11)	0.0031 (12)
C5	0.0330 (14)	0.0352 (14)	0.0277 (12)	−0.0151 (11)	0.0207 (11)	−0.0086 (10)
C6	0.0349 (14)	0.0362 (14)	0.0367 (13)	−0.0151 (11)	0.0258 (12)	−0.0083 (11)
C7	0.0383 (14)	0.0263 (12)	0.0321 (12)	−0.0085 (10)	0.0248 (12)	−0.0065 (9)
C8	0.0326 (13)	0.0286 (13)	0.0275 (12)	−0.0062 (10)	0.0194 (11)	−0.0049 (9)
C9	0.0309 (14)	0.0451 (16)	0.0473 (16)	−0.0033 (12)	0.0220 (13)	−0.0020 (13)
C10	0.0466 (19)	0.055 (2)	0.059 (2)	0.0159 (16)	0.0252 (17)	0.0103 (16)
C11	0.071 (2)	0.0382 (17)	0.057 (2)	0.0114 (15)	0.0393 (19)	0.0134 (14)
C12	0.0588 (19)	0.0316 (14)	0.0473 (16)	−0.0070 (13)	0.0363 (16)	−0.0002 (12)
C13	0.0273 (14)	0.0576 (18)	0.0448 (16)	0.0071 (13)	0.0219 (13)	0.0155 (14)
C14	0.0337 (15)	0.0472 (17)	0.0424 (15)	0.0055 (12)	0.0242 (13)	0.0125 (12)
C15	0.0421 (17)	0.0460 (17)	0.0500 (17)	0.0172 (13)	0.0264 (14)	0.0216 (13)
C16	0.0298 (16)	0.113 (3)	0.0529 (19)	0.0232 (18)	0.0248 (15)	0.010 (2)
C17	0.0267 (15)	0.0587 (19)	0.0532 (18)	−0.0054 (13)	0.0216 (14)	−0.0030 (14)
N1	0.0226 (10)	0.0413 (12)	0.0333 (11)	0.0050 (9)	0.0157 (9)	0.0085 (9)
N2	0.0240 (11)	0.0664 (17)	0.0381 (12)	0.0166 (11)	0.0169 (10)	0.0157 (11)
O1	0.0446 (12)	0.0513 (12)	0.0427 (11)	−0.0287 (9)	0.0276 (10)	−0.0154 (9)
O2	0.0366 (11)	0.0469 (12)	0.0466 (11)	−0.0093 (8)	0.0245 (9)	0.0115 (9)
O1W	0.0269 (14)	0.0260 (13)	0.0350 (13)	0.000	0.0227 (12)	0.000
O3	0.0944 (19)	0.103 (2)	0.0678 (15)	−0.0748 (16)	0.0690 (15)	−0.0539 (14)
O4	0.0261 (9)	0.0332 (9)	0.0295 (8)	−0.0109 (7)	0.0185 (7)	−0.0068 (7)
Cd1	0.01994 (10)	0.02484 (11)	0.02462 (10)	0.00131 (6)	0.01409 (8)	0.00274 (6)

### Geometric parameters (Å, °)

**Table:**

C1—O2	1.245 (3)	C13—H13	0.9300
C1—O1	1.256 (3)	C14—C15	1.351 (4)
C1—C2	1.511 (3)	C14—N1	1.366 (3)
C2—C3	1.370 (3)	C14—H14	0.9300
C2—C8	1.427 (3)	C15—N2	1.357 (4)
C3—C4	1.403 (3)	C15—H15	0.9300
C3—H3	0.9300	C16—N2	1.468 (3)
C4—C5	1.364 (4)	C16—C17	1.474 (4)
C4—H4	0.9300	C16—H16A	0.9700
C5—C7	1.426 (4)	C16—H16B	0.9700
C5—C6	1.503 (3)	C17—C17^i^	1.502 (5)
C6—O3	1.236 (3)	C17—H17A	0.9700
C6—O4	1.258 (3)	C17—H17B	0.9700
C7—C8	1.418 (3)	O1—Cd1^ii^	2.2344 (17)
C7—C12	1.421 (4)	O1W—Cd1^ii^	2.2995 (14)
C8—C9	1.414 (4)	O1W—HW11	0.76 (3)
C9—C10	1.363 (4)	O4—Cd1^iii^	2.3096 (16)
C9—H9	0.9300	O4—Cd1^iv^	2.4848 (15)
C10—C11	1.396 (5)	Cd1—N1	2.264 (2)
C10—H10	0.9300	Cd1—O2	2.2746 (17)
C11—C12	1.351 (5)	Cd1—O1^ii^	2.2344 (17)
C11—H11	0.9300	Cd1—O4^iii^	2.3096 (16)
C12—H12	0.9300	Cd1—O4^v^	2.4847 (15)
C13—N1	1.319 (3)	Cd1—O1W	2.2995 (14)
C13—N2	1.332 (4)		
			
O2—C1—O1	127.1 (2)	C14—C15—H15	126.6
O2—C1—C2	116.9 (2)	N2—C15—H15	126.6
O1—C1—C2	116.1 (2)	N2—C16—C17	113.7 (3)
C3—C2—C8	119.3 (2)	N2—C16—H16A	108.8
C3—C2—C1	119.0 (2)	C17—C16—H16A	108.8
C8—C2—C1	121.6 (2)	N2—C16—H16B	108.8
C2—C3—C4	121.5 (2)	C17—C16—H16B	108.8
C2—C3—H3	119.3	H16A—C16—H16B	107.7
C4—C3—H3	119.3	C16—C17—C17^i^	114.3 (3)
C5—C4—C3	120.3 (2)	C16—C17—H17A	108.7
C5—C4—H4	119.8	C17^i^—C17—H17A	108.7
C3—C4—H4	119.8	C16—C17—H17B	108.7
C4—C5—C7	120.2 (2)	C17^i^—C17—H17B	108.7
C4—C5—C6	120.4 (2)	H17A—C17—H17B	107.6
C7—C5—C6	119.0 (2)	C13—N1—C14	105.2 (2)
O3—C6—O4	124.4 (2)	C13—N1—Cd1	124.23 (18)
O3—C6—C5	115.0 (2)	C14—N1—Cd1	129.70 (17)
O4—C6—C5	120.5 (2)	C13—N2—C15	106.8 (2)
C8—C7—C12	119.2 (2)	C13—N2—C16	126.7 (3)
C8—C7—C5	119.0 (2)	C15—N2—C16	126.4 (3)
C12—C7—C5	121.7 (2)	C1—O1—Cd1^ii^	138.66 (17)
C9—C8—C7	118.2 (2)	C1—O2—Cd1	132.78 (16)
C9—C8—C2	122.5 (2)	Cd1—O1W—Cd1^ii^	114.29 (11)
C7—C8—C2	119.2 (2)	Cd1—O1W—HW11	101 (2)
C10—C9—C8	120.5 (3)	Cd1^ii^—O1W—HW11	115 (2)
C10—C9—H9	119.8	C6—O4—Cd1^iii^	125.42 (15)
C8—C9—H9	119.8	C6—O4—Cd1^iv^	124.57 (15)
C9—C10—C11	121.3 (3)	Cd1^iii^—O4—Cd1^iv^	107.96 (6)
C9—C10—H10	119.4	O1^ii^—Cd1—N1	173.89 (7)
C11—C10—H10	119.4	O1^ii^—Cd1—O2	89.23 (7)
C12—C11—C10	120.1 (3)	N1—Cd1—O2	84.66 (7)
C12—C11—H11	119.9	O1^ii^—Cd1—O1W	92.34 (7)
C10—C11—H11	119.9	N1—Cd1—O1W	87.83 (7)
C11—C12—C7	120.7 (3)	O2—Cd1—O1W	89.30 (6)
C11—C12—H12	119.7	O1^ii^—Cd1—O4^iii^	89.03 (6)
C7—C12—H12	119.7	N1—Cd1—O4^iii^	93.33 (7)
N1—C13—N2	111.8 (3)	O2—Cd1—O4^iii^	114.98 (7)
N1—C13—H13	124.1	O1W—Cd1—O4^iii^	155.70 (5)
N2—C13—H13	124.1	O1^ii^—Cd1—O4^v^	94.20 (7)
C15—C14—N1	109.4 (2)	N1—Cd1—O4^v^	91.89 (7)
C15—C14—H14	125.3	O2—Cd1—O4^v^	172.29 (6)
N1—C14—H14	125.3	O1W—Cd1—O4^v^	83.67 (5)
C14—C15—N2	106.8 (2)	O4^iii^—Cd1—O4^v^	72.04 (6)

Symmetry codes: (i) −*x*, *y*, −*z*−1/2; (ii) −*x*+1, *y*, −*z*+1/2; (iii) −*x*+1/2, −*y*+1/2, −*z*; (iv) *x*−1/2, *y*−1/2, *z*; (v) *x*+1/2, *y*+1/2, *z*.

### Hydrogen-bond geometry (Å, °)

**Table:**

*D*—H···*A*	*D*—H	H···*A*	*D*···*A*	*D*—H···*A*
O1W—HW11···O3^v^	0.76 (3)	1.80 (3)	2.549 (2)	169 (3)

Symmetry codes: (v) *x*+1/2, *y*+1/2, *z*.
